# Application of Ion Mobility Spectrometry for Permeability Studies of Organic Substances through Polymeric Materials

**DOI:** 10.3390/molecules25132983

**Published:** 2020-06-29

**Authors:** Monika Wiśnik-Sawka, Edyta Budzyńska, Jarosław Puton

**Affiliations:** Faculty of Advanced Technologies and Chemistry, Military University of Technology, ul. gen. Sylwestra Kaliskiego 2, 00-908 Warsaw 46, Poland; monika.wisnik@wat.edu.pl (M.W.-S.); budzynska.edyta@wat.edu.pl (E.B.)

**Keywords:** diffusion in polymers, permeability, tubular membrane, extraction from water, ion mobility spectrometry

## Abstract

Drift tube ion mobility spectrometers (DT IMS) allow the concentration of different organic compounds to be measured. This gives the opportunity to use these detectors in measuring the penetration of various substances through polymer membranes. Permeation measurements of two substances (2-heptanone and dimethyl methylphosphonate (DMMP)) through a cylindrical silicone rubber membrane were carried out. The membrane separated the aqueous solution from the air. The analyte was introduced into water, and then its concentration in air on the opposite side of the membrane was recorded. Based on the dynamics of detector signal changes, the diffusion coefficients for both tested substances were determined. Determination of permeability coefficients was based on precise quantitative measurements, which took into account the non-linearity of the detector characteristics and the effect of water on detection sensitivity. The analysis of measurement results was based on a mathematical description of diffusion process.

## 1. Introduction

Polymeric materials (PM) are commonly used in technology and everyday human activity. The most important PM performance parameters are durability, elasticity and other mechanical properties. For some applications, properties such as the permeability for some chemicals and their solubility are important. This is particularly crucial for polymer packaging and protective coatings. A specific type of PM applications are membranes used for controlled transport of substances between two phases. They are successfully used in industry [1,2,3], as well as in medicine [4,5] and analytical techniques [6,7].

In the separation technology, membranes are thin layers characterized by permeability and selectivity. High selectivity allows for the enrichment or depletion of a specific component of a mixture. 

There are three basic types of membranes: porous, dense and liquid. Membranes belonging to these groups differ not only in the structure but also in the mechanism of substances’ penetration through them. In the case of porous membranes, separation can occur due to the fact that the particle sizes are comparable with pore sizes. In this case, selective permeation of the substance through the membrane is called the ‘sieve effect’ and can occur according to various mechanisms. For larger pore sizes, molecular diffusion is observed, and for micropores at low pressure, Knudsen diffusion occurs. For dense membranes, the separation of components is determined by the diffusion rate and the solubility of the component in the material. Membranes are made of amorphous polymers. These substances are divided into two groups: rubbery and glassy polymers. The main difference between permeation processes in these materials is due to their structure [8]. In non-crystalline rubbers, there are regions in which the polymer arrangement produces semi-crystalline fragments in the form of chain clusters forming tubules. Transportation of the permeating substance takes place by jumping between tubules. Glassy polymers are characterized by rigid connections between polymer chains. Due to the lower mobility of the chains, the substance is transported between areas not occupied by atoms contained in the polymer macromolecules [9]. This reduces the permeability of larger particles through the material. Permeation of the substance through the described materials is mostly determined by the solubility coefficient. Its value is dependent on the interactions between the membrane material and penetrant molecules. The solubility coefficient significantly affects membrane selectivity [10,11]. It is assumed that the permeation of substances according to this mechanism performs in several stages. Initially, the particles of the substance are adsorbed on the surface of the membrane. The next stages are the dissolution of the substance in the polymer and its diffusive transport. The permeation process ends with desorption on the opposite side of the membrane.

Determination of the parameters defining the transport of substances through membranes can be done using several experimental methods. For dense membranes, flow methods are usually used. In these methods the penetrant mass flow is measured. Sorption methods based on the absorption/desorption of a substance in/from a membrane are used much less frequently. Determination of mass flux in flow methods can be carried out in various ways—starting from measuring the pressure value and ending with the use of analytical techniques—to determining the amount of permeating substance. The traditional way of testing gas permeation through polymer membranes is based on the pressure method. In order to perform such tests, a membrane of known thickness is installed in a tight chamber. The permeation process is forced by the pressure difference between the two sides of the membrane. After creating the appropriate initial pressure difference, an increase (or decrease) of the pressure is observed on one side of the membrane [12]. Hereby, the amount of permeating gas [13,14] or the flux value [15] is determined.

Studying of substance transport through membranes can be conducted using different detectors used in chemical analysis. These types of methods are suitable when permeation occurs under the influence of pressure differences, as well as when diffusion flow is caused by a difference in concentration on both sides of the membrane. The measurement of CO_2_ permeation through polymer coatings can be performed with a typical analyzer operating on the basis of infrared absorption [16,17]. For the determination of oxygen permeability through membrane, the value of the oxygen flux can be measured by polarographic system [13,18]. In studies of the permeation of organic substances through polymer membranes, chromatographic techniques are used [19,20,21,22,23,24,25,26]. Introduction of samples into the chromatograph can be made using different methods. Chromatograph can be often coupled with various detectors such as mass spectrometers [21,23,24] or electron capture detectors [22]. These measurements are performed using cyclic sampling. For this purpose, the non-direct connection of the chromatograph to the permeability measurement system (off-line methods) is usually used. However, when specific sample introduction systems are used, it is possible to directly connect a chromatograph with a diffusion cell (on-line methods) [25,27]. The application of on-line systems enables full automation of the experimental process.

This paper contains the results of flow experiment performed using an ion mobility spectrometer. The ion mobility spectrometry (IMS) has been applied for the past 50 years and successfully used as an analytical method for the detection and identification of large number of chemical compounds [28,29,30,31]. Polymer membranes are used in sample introduction systems for IMS detectors. In published works [32,33,34,35,36,37,38] it has been shown that inlet systems with a polymer membrane provide effective detection of analytes present in the gas and liquid phases. The purpose of our work is not to examine the effectiveness of detection, but to determine the parameters characteristic for diffusion process. In our experiment the IMS detector was used as a device for measuring the mass flux of the permeating substance. The selection of this detector for conducting mass flux measurements was due to high sensitivity (detection limits below 1 ppb), selectivity and usefulness for detection of various compounds. IMS detectors are devices characterized by fast analytical signal generation (time constant of a few seconds) and the possibility of continuous operation. Our measurements were made for a hollow cylinder silicone membrane that separated the analyte-containing water solution from the air.

## 2. Theory

Penetration of the substance through dense polymeric materials occurs according to the aforementioned dissolution mechanism. The diffusion process determines the time-dependent distribution of the penetrating substance concentration in the polymeric material. In the simplest case, for homogeneous flat layers made of a dense polymer, the mass flow of the penetrant can be described by the first Fick diffusion law:

(1) $$F=-D\frac{\partial C}{\partial x}$$

where *F* is the penetrant flux density, defined as the amount of substance flowing through the surface unit in the time of 1 second, *D* is the diffusion coefficient, and *C* is the concentration of the penetrant in the membrane. In general, the concentration *C* and the flux density *F* are time dependent values. In steady state, for a membrane with a thickness of *l*, the flux density *F_ST_* of the substance penetrating through the membrane is described by the formula:

(2) $$F_{ST}=D\frac{\left(C_{0}-C_{l}\right)}{l}$$

where *C_0_* and *C_l_* are the penetrant concentrations in the membrane material on both sides of it. They are linearly dependent on the concentrations of *C_0,ext_* and *C_l,ext_* in the environments separated by the membrane, with the proportionality constant, which is the solubility *S*, being the measure of the ability of the polymer for penetrant absorption:

(3) $$\begin{array}{l} C_{0}=SC_{0,ext}\\ C_{l}=SC_{l,ext} \end{array}$$

The permeability *P* of a given membrane material for given penetrant is defined as the amount of substance penetrating per area unit of the membrane with a unit thickness in 1 second, related to the difference in concentration in the media separated by the membrane:

(4) $$P=\frac{F_{ST}l}{\left(C_{0}{}_{,ext}-C_{l,ext}\right)}$$

It follows from formulas (2), (3) and (4) that the permeability is a product of diffusion coefficient *D* and solubility *S*:

(5) P=DS

Determination of the permeability coefficient based on the formula (4) can be performed for the equilibrium state. In this case, it is necessary to know the dimensions of the membrane, the value of the diffusion flux and the concentration values on both sides of the membrane. Determination of the diffusion coefficient can be made only on the base of the results obtained from dynamic measurements.

The most commonly used for considerations of the diffusion process is the assumption that the polymeric material is isotropic. Diffusion in such material occurs equally in all directions. Theoretical determination of concentration distribution is possible by solving the equation describing the second diffusion law:

(6) $$\frac{\partial C}{\partial t}=\mathrm{div}(D\;\mathrm{grad}\;C)$$

**Figure 1:**
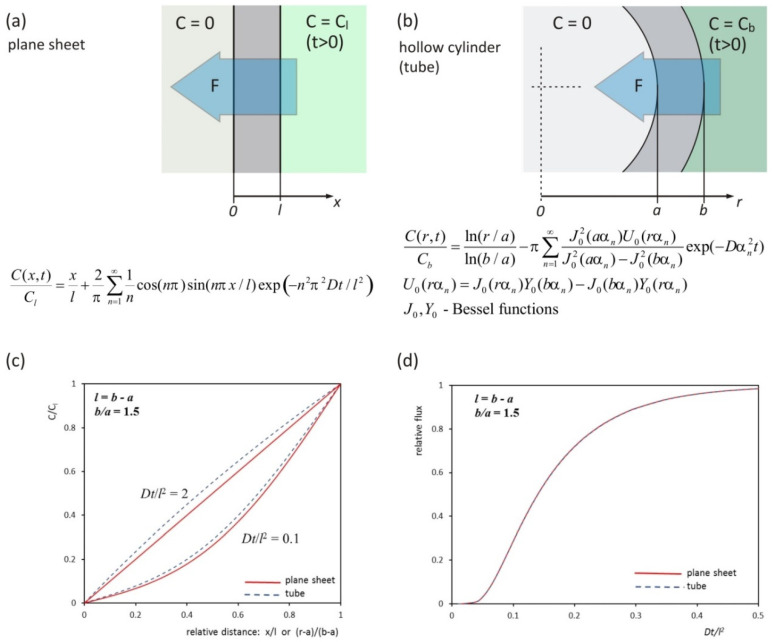
Diffusion transport in flat and hollow cylindrical membranes. Sketches of transport geometry through membranes with formulas describing concentration distribution for flat (**a**) and hollow cylindrical (**b**) membranes. Comparison of penetrant concentration distributions in a flat and hollow cylindrical membranes (**c**). The dependence of the mass flow of the substance diffusing through the membranes on the time (**d**).

Solutions of this equation for different geometries can be found in many publications [39,40,41]. Sketches illustrating the process of penetration through a flat and hollow cylindrical membrane are presented in Figure 1a,b. Both cases are one-dimensional. The drawings also include formulas describing concentration distributions for zero initial condition and boundary conditions corresponding to constant concentration values on the edges. Shapes of the concentration distribution for flat and cylindrical membranes are different (Figure 1c). In order to determine the diffusion coefficient in flow experiments, the dependence of the diffusing substance mass flux on time is used. Values of flux at x = 0 for a flat membrane and r = a for a hollow cylindrical membrane were calculated by numerical differentiation of concentration distributions. The dependence of the diffusion flux shape on time for flat and hollow cylindrical membranes are shown in Figure 1d. These shapes are almost identical in both cases. This means that the flow experiment results obtained experimentally for a hollow cylindrical membrane can be carried out based on the formulas resulting from the mathematical model for a flat membrane.

The analysis of the mass flux dependency of the penetrant through the membrane is often made using the so-called short-time approximation method. It is based on the solution of the diffusion Equation (6) with the use of Laplace transform [39,40,41,42]. For short times it is possible to present the solution for mass flux density in the form:

(7) $$\ln\left(t^{\frac{1}{2}}F\right)=\ln\left\{2C_{l}{\left(\frac{D}{\pi }\right)}^{\frac{1}{2}}\right\}-\frac{l^{2}}{4Dt}$$

The application of formula (7) to data obtained from the measurement of the mass flux allows the determination of the diffusion coefficient. It is crucial for determination of maximum value of the flux. Using formula (4) and knowing the maximum value of the flux *F_ST_* in steady state, one can determine the value of permeability.

## 3. Materials and Methods

### 3.1. Chemicals

Permeability tests were performed for a commercially available silicone tube—SILASTIC^®^ Laboratory Tubing (Dow Corning, Midland, MI, USA)—made of translucent, platinum hardened, tear resistant silicone. This type of tube is used in laboratory systems requiring flexible and temperature resistant connections, as well as where high purity of fluids flowing through the tubes is of great importance. Tubes with an outer diameter of 0.318 cm, an inner diameter of 0.198 cm and a length of 32 cm were used in this study.

Measurements of permeability and diffusion coefficient were made for two organic compounds: 2-heptanone (Sigma-Aldrich, Saint Louis, MO, USA, 99% purity) and dimethyl methylphosphonate (DMMP) (Alpha Aesar, GmbH, Germany, 97% purity). Water solubilities of these compounds are about 0.4 g/100 ml and about 10 g/100 ml, respectively. Water used in the tests was purified in the Hydrolab HLP5UV filter system (Wenk Labtec, Germany). The gas flowing inside the tube was air purified in a filter of 2000 cm^3^ volume filled with molecular sieves with 1.0 nm pores diameter (Merck, Germany).

### 3.2. Instrumental

**Figure 2:**
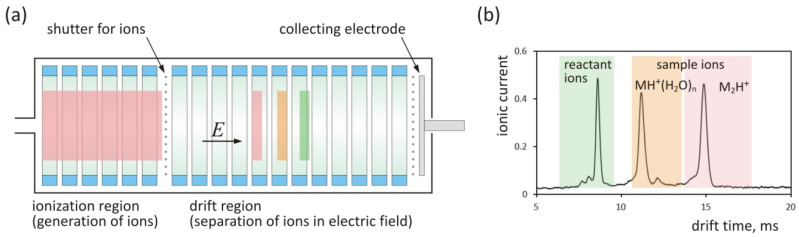
Sketch of the drift tube ion mobility spectrometer (DT IMS) design (**a**). Exemplary drift time spectrum containing reactant ions H_3_O^+^ (H_2_O)_m_, monomer ions MH^+^(H_2_O)_n_ and dimer ions M_2_H^+^ (**b**).

The detector used for measuring the flux of the substance permeating through the membrane was a drift tube ion mobility spectrometer (DT IMS). The operation principle of this detector is illustrated in Figure 2a. The detector consists of two parts: an ionization region in which ions are formed and a drift section where separation of ions occurs. Both parts are separated by a shutter grid that allows the narrow portion of ions to be introduced into the drift section. Two gas streams are introduced into the detector: carrier gas, which contains the sample, and drift gas. In most cases, purified air is used as the carrier gas and drift gas. The output signal from the DT IMS collector electrode (Figure 2b) is called the drift time spectrum and contains information about mobility of ions and their quantity. The spectrum consists of peaks generated by reactant ions and sample ions. Reactant ions are formed as a result of ionization of the carrier gas and are a reservoir of electric charge transferred to the sample molecules. For most substances, monomer and dimer ions are formed. The exemplary spectrum presented in Figure 2b contains peaks of reactant ions as well as the peak of hydrated monomer (MH^+^(H_2_O)_n_) and dimer (M_2_H^+^) ions. In quantitative studies, peak areas are most often measured. Determination of analyte concentration can be based on the area of reactant ions peaks or peaks generated by sample ions.

The DT IMS used in the study was designed and manufactured at the Institute of Chemistry, Military University of Technology in Warsaw. The detector is equipped with 63-Ni ionization source with an activity of approximately 15 MBq, which is placed in ionization region. Its length is 5.7 cm. The ionization region is separated from the drift section by a Bradbury-Nielsen shutter grid opened for a time of 0.150 ms with a repetition period of 25 ms. The drift section is 6.1 cm long, and the electric field in this section is equal to about 250 Vcm^−1^. The inner diameter of the detector is equal to 3.6 cm. Laboratory air dried with molecular sieves with a pore diameter of 1 nm (Merck) was used as the carrier and drift gases.

**Figure 3:**
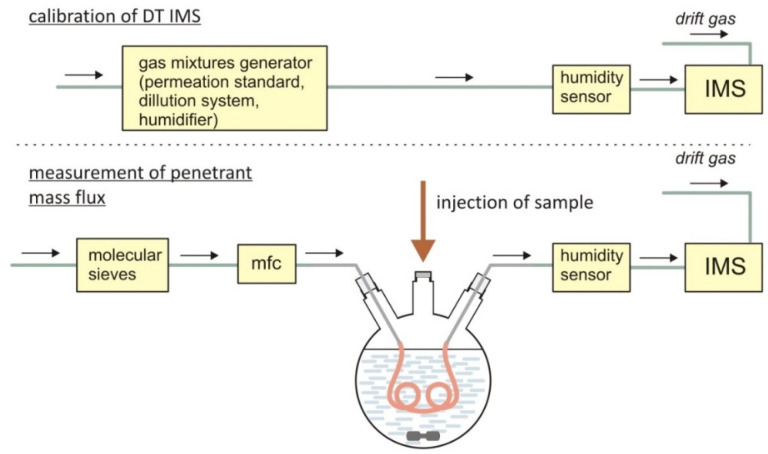
Scheme of the experimental system for testing the permeation of substances contained in water into the air through cylindrical silicone membranes (mfc—mass flow controller).

The scheme of the system used for the measurement of the flux of substance permeating through a hollow cylinder membrane is shown in Figure 3. The design of this system is similar to that presented in the work by Du [33]. The basic element of the system was a reaction kettle filled with water, inside which the tested tube was placed. The water temperature in the kettle was kept at 25 °C. By means of mass flow controller (mfc), 0.7 Ln/min of gas was introduced into the tube. The polymer membrane (silicone tubing) was submerged in water, into which the analyzed substance was injected and mixed with a magnetic stirrer. The air passing through the tube was directed to the IMS detector via the DPT-21 humidity sensor (Czaki Thermo-Product). The analytical signal obtained from IMS detectors is strongly related to the water vapor content in analyzed gas. It significantly affects the ionization process. This involves the formation of hydrated cluster ions with a higher number of water molecules, which leads to the reduction of ionization efficiency. It has been proven [43,44] that with the increase of humidity there is a simultaneous decrease in peaks amplitude and a shift towards higher drift times. Due to these phenomena, it is necessary to control the water vapor concentration in the carrier gas.

The measurement of the diffusion flux began when the analyte had been injected into the water filling the reaction kettle. From this moment, drift time spectra were recorded at 4 min intervals. The tests were quantitative, and therefore, it was necessary to perform calibration measurements of the detector first. A gas mixtures generator was used in calibration studies. The generator system enabled the introduction of the analyte with a concentration of 0–20 ppb for 2-heptanone or 0–12 ppb for DMMP into the carrier gas. The mixture produced in the generator was moisturized in order to obtain the same water vapor content as during permeability tests.

## 4. Results

**Figure 4:**
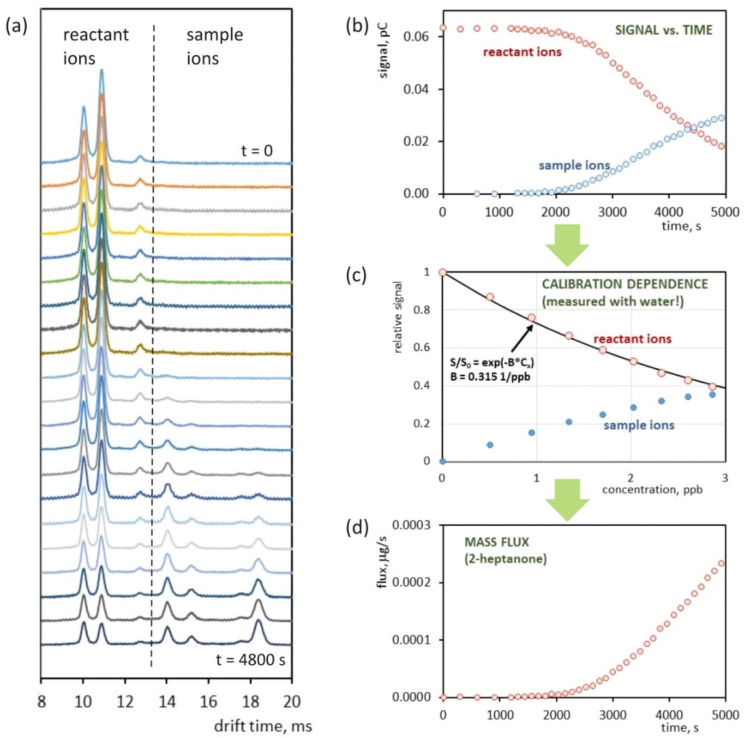
Determination of 2-heptanone mass flux through the cylindrical silicone membrane. Drift time spectra recorded after 2-heptanone injection in 4-minute intervals (**a**). Dependence of the IMS detector signal intensity on time measured for reactant ions and sample ions (**b**). 2-heptanone calibration curve determined at a water concentration of 226 ppm (**c**). Mass flux of 2-heptanone permeating through the polymer membrane calculated on the basis of IMS signal and calibration curve (**d**).

The mass flux of the substance permeating through the membrane was measured for two organic substances: 2-heptanone and dimethyl methylphosphonate (DMMP). Figure 4a presents drift time spectra recorded for 2-heptanone at different times from the introduction of the sample into the water in which the silicone tube was placed. The subsequent spectra show the appearance of analyte ion peaks and change in their amplitudes. The intensity of the reactant ions peaks also changes (decreases). The relationship between the peak areas and time is shown in Figure 4b. The numerically determined peak area of the reactant ions peaks was taken as a measure of the IMS detector signal and used for the calculation of the parameters characterizing the permeation through the membrane. The calibration curve measured with the gas mixtures generator is shown in Figure 4c. Measurements were made at the same water vapor content as during permeability tests. The mass flux dependence on time shown in Figure 4d was calculated on the basis of changes in the detector signal in time and the calibration curve. In this calculation, the value of the gas flow through the tube was taken into account.

**Figure 5:**
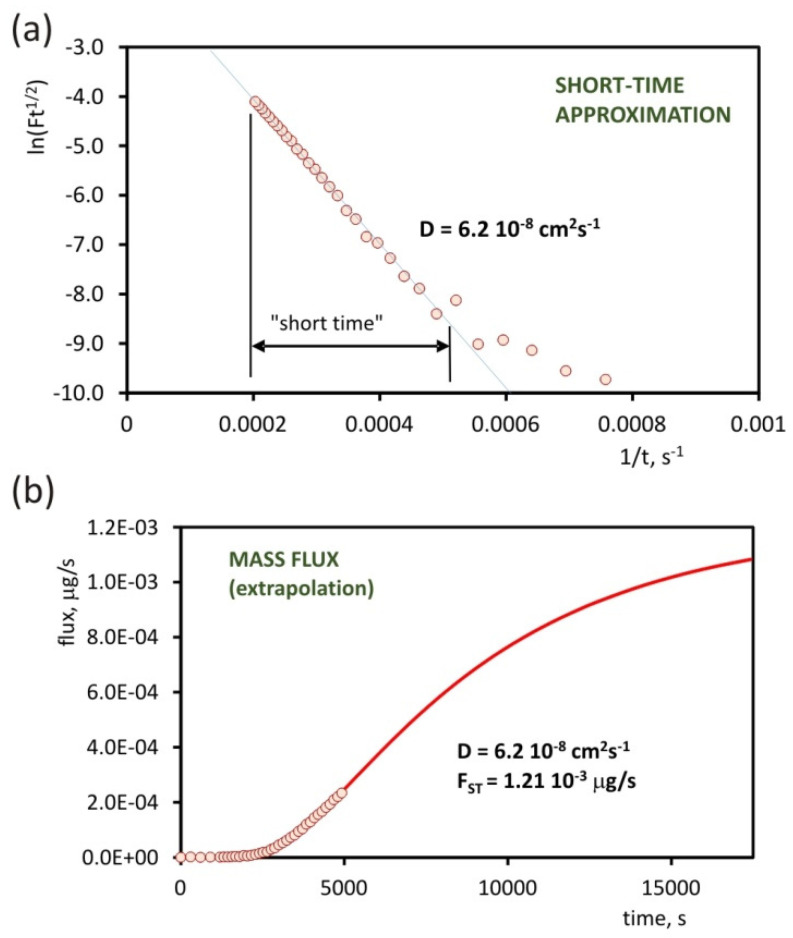
Graph of dependence ln(*Ft*^1/2^) from 1/*t* used for determination of the diffusion coefficient by the short time approximation method (**a**). Extrapolated mass flux versus time, determined on the basis of the calculated diffusion coefficient (**b**).

Determination of the diffusion coefficient for 2-heptanone was carried out using the short time method. Figure 5a is a graph of the dependence between ln(*Ft*^1/2^) and 1/*t* calculated on the basis of mass flow measurement results. For times from 2000 to 5000 seconds, this dependence is linear. Based on formula (7), the diffusion coefficient was determined using the slope of the linear part of this dependence. For 2-heptanone it was 6.2 × 10^−8^ cm^2^s^−1^. The determined *D* value allows to calculate the theoretical mass flow (Figure 5b) and estimate its value for equilibrium state *F_ST_*. Basing on this value and using the formula (4), it is possible to calculate permeability *P*. Its value for 2-heptanone is 2.0 × 10^−7^ cm^2^s^−1^. The results for the second analyte (DMMP) were analyzed analogously. 

The accuracy of determining the diffusion coefficient is related to the precision of reproducing the shape (but not the amplitude) of the permeation curve. We estimate that the relative uncertainty of the calculated diffusion coefficient is no more than 20%. The uncertainty of determining the permeability coefficient is mainly determined by the accuracy of measuring the concentration of the analyte in the gas flowing inside the tube. The value of this uncertainty seems not to be greater than 10%.

Estimation of permeability and diffusion coefficient were also carried out for water. The water flux was measured using a capacitive humidity sensor. Measurement of water concentration in the air flowing through the tube allowed the permeability to be determined. Its value was equal to 4.3 × 10^−9^ cm^2^s^−1^. The diffusion coefficient for water was estimated based on the results of dynamic measurements. They involved the observation of changes in the water concentration in the air flowing through the tube after water was introduced into the reaction kettle. The shape of the dependence of water vapor concentration on time was similar to the curve shown in Figure 5b. Maximum value of water vapor concentration was equal to 230 ppm. This value was constant for times longer than 2000 s after introducing the water to the kettle. The value of water vapor concentration equal to half of the steady state value was achieved after approximately 400 s. Based on theoretical considerations, it can be easily demonstrated that this corresponds to a *Dt/l*^2^ value of 0.15, which, with the known time and membrane thickness, gives a diffusion coefficient value of 1.4 × 10^−6^ cm^2^s ^−1^. It can be assumed that this value is underestimated due to the quite significant time constant of the humidity sensor. All values of diffusion and permeability coefficients determined on the basis of the measurements carried out are summarized in Table 1.

## 5. Conclusions

Drift tube ion mobility spectrometer give the opportunity to measure the concentration of many organic compounds. This feature can be used in studies of the organic compounds’ permeation through polymer materials.

The aim of the presented work was to test a new measurement system for studying the permeation of organic substances through polymer membranes. Until now, similar systems have been used only for analytical purposes, i.e., for checking if such membranes can be used for extracting the analyte from water. [43,44]. In our work, parameters describing the transport of chemicals in polymers, i.e., diffusion coefficients and permeability, have been determined. One should realize that in our studies the membrane separated the liquid phase from the gas. This has a significant impact on mass transport conditions. The obtained values of organic substances diffusion coefficients are relatively small. This may be due to the surface interaction of the penetrant with the polymer layer or to the fact that the polymer material is saturated with water. Relating the obtained results to literature data is difficult because the types of silicone rubber and research conditions are very different. The diffusion coefficient of methyl ethyl ketone (butanone) in the silicon membrane determined by Thiyagarajan et al. was 7.6 × 10^−6^ cm^2^s^−1^ (at 40 °C) [45]. This is over 100 times more than the value determined based on our research. However, it should be noted that our measurements were made at a lower temperature, heptanone particles are much larger than butanone and that the structure (fillers, degree of cross-linking) of the polymer material was different. Water permeability is much lower than for organic substances. This is advantageous for membranes separating the DT IMS reaction area from the environment, as it limits the water concentration in the carrier gas. This allows the ionization efficiency of many analytes to be increased.

The concentration of organic substances in the aqueous solution was low (about 10 μg/g); however, with the use of a polymer membrane, effective detection was possible. This confirms the usefulness of silicone membranes in analytical applications, since they can be used to separate the reaction area of the detector from the environment.

## Figures and Tables

**Table 1:** Determined values of diffusion coefficient and permeability of 2-heptanone, DMMP and water in silicone rubber.

	Diffusion Coefficient	Permeability
2-heptanone	6.2 × 10^−8^ cm^2^s^−1^	2.0 × 10^−7^ cm^2^s^−1^
DMMP (dimethyl methylphosphonate)	3.1 × 10^−8^ cm^2^s^−1^	4.9 × 10^−8^ cm^2^s^−1^
water	>1.4 × 10^−6^ cm^2^s^−1^	4.3 × 10^−9^ cm^2^s^−1^
